# Mechanically Optimize T Cells Activation by Spiky Nanomotors

**DOI:** 10.3389/fbioe.2022.844091

**Published:** 2022-02-22

**Authors:** Dongmei Fu, Dazhi Xie, Fei Wang, Bin Chen, Zhen Wang, Fei Peng

**Affiliations:** ^1^ School of Materials Science and Engineering, Sun-Yat-sen University, Guangzhou, China; ^2^ School of Pharmaceutical Science, Southern Medical University, Guangzhou, China

**Keywords:** nanomotors, mechanical force, T cells activation, mechanosensitive ion channels, spiky

## Abstract

T cell activation is vital for immune response initiation and modulation. Except for the strength of the interaction between T cell receptors (TCR) and peptides on major histocompatibility complex molecules (MHC), mechanical force, mediated by professional mechanosensitive ion channels, contributes to activating T cells. The intrinsic characteristic of synthetic micro/nanomotors that convert diverse energy sources into physical movement and force, opening up new possibilities for T cell regulation. In this work, Pd/Au nanomotors with spiky morphology were fabricated, and in the presence of low concentrations of hydrogen peroxide fuel, the motors exhibited continuous locomotion in the cellular biological environment. Physical cues (force and pressure) generated by the dynamic performance are sensed by mechanosensitive ion channels of T cells and trigger Ca^2+^ influx and subsequent activation. The successful demonstration that mechanical signals generated in the bio microenvironment can potentiate T cells activation, represents a potential approach for cell-based cancer immunotherapy.

## Introduction

A micro/nanomotor is a biomimetic system of natural molecular motors and micro-organisms that converts energy into movement and force (Abdelmohsen et al., 2014). Typically, to propel in biological media, these artificial machines rely on either chemically powered [including hydrogen peroxide (Wilson et al., 2013), glucose (Ma et al., 2015), urea (Hortelão et al., 2018), etc.] or external energy sources [such as light (Ibele et al., 2009), ultrasonic (Lu et al., 2019) or magnetic fields (Liu et al., 2020), etc.]. Such unbound tiny machines have inherent advantages such as active transport, high tissue penetration, and motion controllability, indicating immense potentials for a variety of biomedical applications in targeted drug/cell delivery (Tu et al., 2017a; Tu et al., 2017b) minimally invasive surgery (Malachowski et al., 2014; He et al., 2016) and biosensing (Molinero-Fernandez et al., 2020), etc., serving as a revolutionary toolbox for cancer diagnosis and therapy.

In recent years, immunotherapy has created a novel paradigm for cancer treatment and has made many extraordinary breakthroughs in clinic practice (Mellman et al., 2011). Among them, activation of T cells is a key step in cell-based immunotherapies. Generally, T cells are activated in response to the interaction of T cell receptors (TCRs) with peptides on major histocompatibility complex molecules (pMHC)and induction of downstream signaling (Restifo et al., 2012). Benefiting from the signal transduction of the immune cascade, methods have been developed to activate T cells with various cytokines (IL-6, IFN-*γ*, CXCL10, etc.). While effective treatment, it also faces the risk of causing excessive activation of T cells and eventually leading to a cytokine storm. To reduce this risk, precise local activation methods are required. The micro/nanomotor system has advantages in temporal and spatial controllability, making it a potential candidate for local activation of T cells. To modulate immune cells, current works have loaded micro/nanomotors with various antigens and stimulatory ligands and used them as artificial antigen-presenting cells (APCs). For instance, Lee et al. (Lee et al., 2016) achieved remote activation of T cells using Janus magnetic particles with anti-CD_3_ coating. Our group (Wang et al., 2020) proposed a magnesium (Mg)-based polymeric micromotor system loaded with chemical stimulators, as an additional biocompatible system for immune cell regulation. However, these immune cells regulation focus on agonists modified on the carrier, bypassing the innate mechanical performance caused by the motility itself as well the need for mechanical cues in T cell activation. Actually, in addition to the strength of the interplay of TCRs with pMHC, mechanical forces and pressure contribute to accentuating T cell activation *via* mechanosensitive cation channels, especially Piezo1 members (Liu et al., 2018; Pan et al., 2018; Liu and Ganguly, 2019). Recent data suggest that such mechanosensitive ion channels are widely expressed in human immune cells and function as professional mechanotransducers at the immunological synapse, thus playing a crucial role in cell activation. Solis et al. (Solis et al., 2019) found that sustained mechanical stimulation (cyclical hydrostatic pressure and force) can induce immunity activation, demonstrating that mechanical signals can also be used as regulators of immune cell function. Moreover, by further quantification, mechanosensitive channels of immune cells can be activated with a force near 10 pN (Wu et al., 2016). Accordingly, Ma et al. (Ma F. et al., 2015) used an optical tweezer setup to measure the effective force generated by a single enzyme-driven nanomotor after breaking through the self-thermal force of Brownian motion (∼40 pN in this work). In addition, the continuous self-driven behavior of the motor in the extracellular environment induces rapid convection of the surrounding fluid, and this dynamic microenvironment may have a synergistic effect on the force of the motor acting on the cell (Paxton et al., 2004; Solis et al., 2019; Walmsley, 2019; Lopez-Ramirez et al., 2020). Hence, it is reasonable to use nanomotors to activate T cells because it can deliver physical signals to elicit responses from mechanosensitive ion channels and ultimately induce cell activation.

Although various forms of synthetic micromotors and nanomotors have been developed, with Janus particles, rods, spiral structure, and tube form (Gao et al., 2013; Ma X. et al., 2015; Zhang et al., 2020), the impact of motor morphology on its performance has not been systematically studied so far. Especially, the spiky nanomotors prepared in this study resemble many pathogens with spike-like nanostructures on their surface, which are known to be crucial for their adhesion and infection (Christo et al., 2016). Wang et al. (Wang et al., 2018) demonstrated spiky nanoparticles to activate and amplify the immune response through exerting mechanical stress on the cells and their research sheds light on the significance of nanostructural cues in the regulation of innate immune response, deducing that spiky nanomotors may have an analogous effect on the immune system.

**FIGURE 1:**
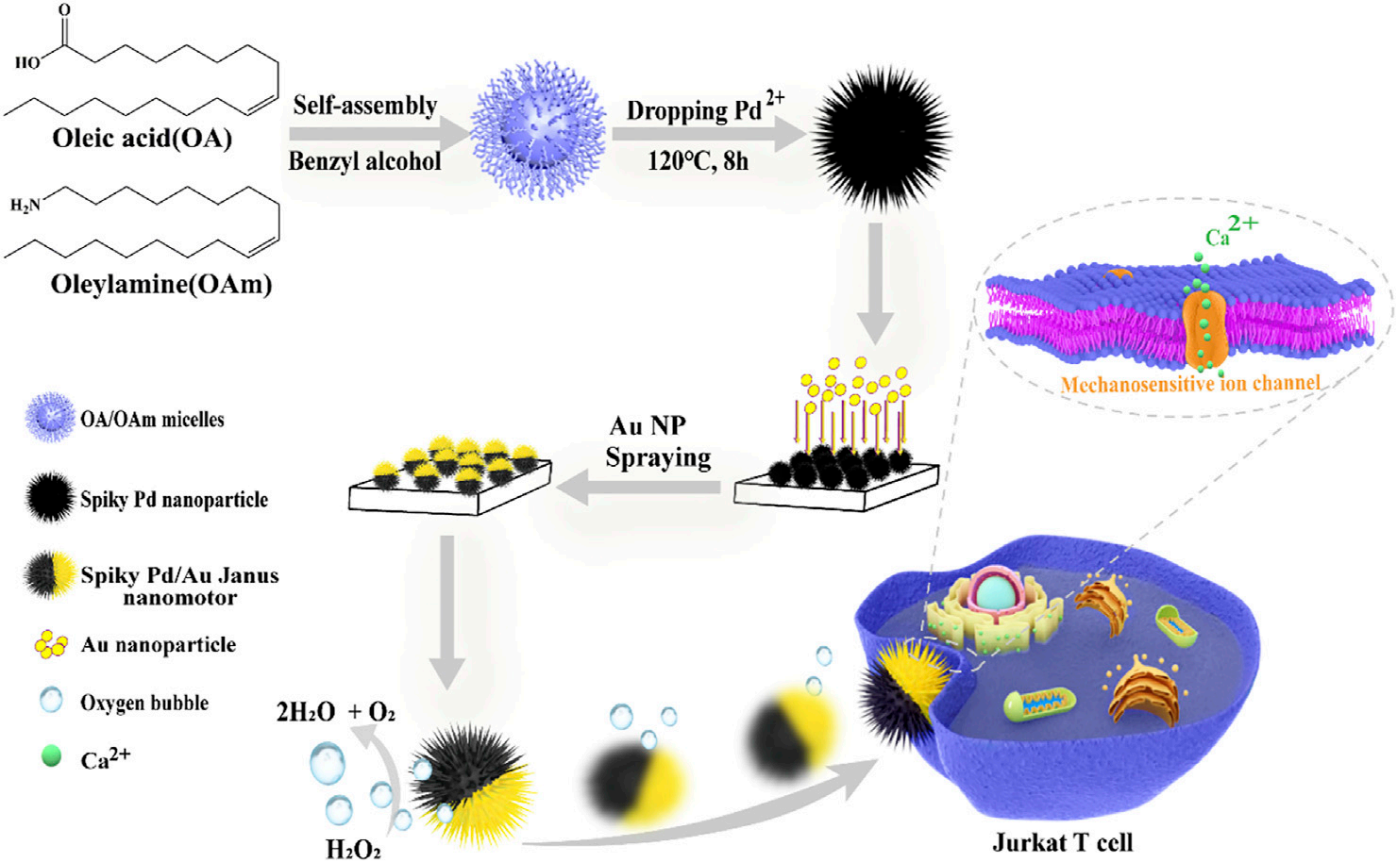
Schematic illustration of the construction process of spiky Pd/Au Janus nanomotors and the fast-moving nanomotor triggers T cell activation mediated by the professional mechanosensitive ion channels.

Herein, we fabricated Pd/Au Janus nanomotors decorated with nano-spikes and utilize the mechanosensitive ion channels (calcium-permeable) as a sensor to investigate their ability for activating T cells *in vitro*. Figure 1 schematically illustrates the synthesis of the spherical Pd nanoparticles with dendric structures *via* hydrothermal reaction, and then asymmetrically sputtering with a thin gold (Au) layer on one side of the nanospheres for the fabrication of nanomotors. By decomposing hydrogen peroxide, the thrust was generated and the motor was pushed forward. Subsequently, the nanomotors fast-moving around T cells or in or induce convection of the environment fluid outside the cell as a whole, and the pressure generated is transmitted to mechanosensitive ion channels, which largely exist on the membrane of T cells, making inwardly flow of calcium ions and eventually optimize T cell activation overall.

## Materials and Methods

### Materials

Palladium chloride (59–60%) was purchased from Macklin Biochemical Co., Ltd. (Shanghai, China), hydrochloric acid (AR), hydrogen peroxide (30%) was purchased from Guangzhou brand reagent company (Guangzhou, China), oleic acid, oleylamine, benzyl alcohol (99%), absolute ethanol, and sucrose were obtained from Aladdin Biochemical Technology Co. (Shanghai, China). Fluo-4 AM, Hoechst 33342, Trypan Blue Solution (0.4%), HEPES buffer solution (pH = 7.4), PBS buffer solution (pH = 7.4) was bought from Beyotime Biotechnology (Shanghai, China). Roswell Park Memorial Institute (RPMI-1640) cell media were bought from Gibco. Jurkat T cells were donated by Southern Medical University. All other chemical reagents used in this experiment were analytically pure without further purification. Purified deionized water was prepared by the Milli-Q Plus system (Millipore, United States).

### Fabrication of Spiky Pd/Au Nanomotors

Pd nanospikes were firstly synthesized by hydrothermal method. In a typical procedure, 0.2 mmol PdCl_2_ (dissolved in 1M HCl), 80.0 ml benzyl alcohol, 4.0 ml oleylamine (OAm) and 4.0 ml oleic acid (OA) were mixed in a 100 ml beaker and stirred at room temperature for 30 min. Then, it was transferred to a stainless teel autoclave lined with a polytetrafluoroethylene container, and reacted at 120°C for 8 h. Naturally cool to room temperature, centrifuge at 10,000 rpm to remove the surfactant, wash the precipitate with ethanol 3 times, and then dry it under vacuum at 40°C. Subsequently, an aqueous solution (∼200 mg/ml) containing Pd nanoparticles was dropped onto the glass slide by a spin coater to form a monolayer, using an ion sputter coater to sputter gold (∼3 nm, 50s) were used to produce Pd/Au Janus surface. Then, it was collected by ultrasound and resuspended in PBS, washed three times, and dispersed in PBS buffer solution for further use.

### Motion Evaluation of Pd/Au Nanomotors

The optical videos were recorded by A Nikon Ti2-A inverted optical microscope with a ×40 objective. Dilute Pd/Au nanomotors to the appropriate concentration with PBS buffer and add H_2_O_2_ solution with different concentrations (0.00, 0.05, 0.10, 0.50% wt, add 10 µL in 1 ml PBS dispersion solution, the final concentration is 0.00, 0.165, 0.33, 1.65mM, respectively) in a petri-dish and record the movement videos up to 10 s at 10 fps, respectively. Then, the moving tracking trajectories and the speed of nanomotors (at least 15) were analyzed by ImageJ.

The mean square displacements (MSD) curves were fitted by the following Eq. 1 (Arque et al., 2019; Tang et al., 2020): $MSD\left(\Delta t\right)=<{\left(x_{i}\left(t+\Delta t\right)-x_{i}\left(t\right)\right)}^{2}$ >, where *i* = 2 refers to two-dimensional analysis.

Extract the slope of the MSD curves, and then calculate the long-term diffusion coefficient (D_L_) according to the formula 2 (Wang et al., 2019; You et al., 2019):

$$MSD=4D_{L}\Delta t$$

### Cell Culture and Staining

Jurkat T cells were cultured in Roswell Park Memorial Institute (RPMI-1640) cell media supplemented with 10% (v/v) fetal bovine serum (FBS) and penicillin/streptomycin (1%, v/v) in an incubator at 37°C with an atmosphere with 5% CO_2_. For calcium imaging, transfer the cells to a serum-free cell culture medium and starve them for 2 h. Then, incubate with 5 μM Fluo-4 AM in serum-free cell culture medium at 37°C for 30 min, and then stain with Hoechst 33342 for 10 min.

### Monitor the Activation of Jurkat T Cells

Resuspended the Jurkat T cells loaded with Fluo-4 AM with the DEP cell medium and seeded into the 96-well plates with a density of about 1× 10^4^ cells per well. Incubated the cells with Pd/Au nanomotors (∼20 μg/ml) in the presence or absence of H_2_O_2_ (add 2 µL 0.05%wt in 200 µL dispersion, final concentration is 0.165 mM) and only H_2_O_2_ were investigated, respectively. Then, the green fluorescence of Fluo 4 was captured by A Nikon Ti2-A fluorescence microscopy (Intensity:100%; Exposure time:100 ms; Gain:1.0X). Corresponding fluorescence intensity was calculated with the Image J software according to the previous research (Sheffield 2007).

### CCK-8 Assay and Live/Dead Staining

The cytotoxicity of nanomotors was assessed by a standard CCK-8 method. NIH3T3 cells were cultured with Dulbecco’s Modified Eagle Medium (DMEM) supplemented with fetal bovine serum (FBS, 10%,v/v) and penicillin/streptomycin (1%, v/v) at 37°C in a 5% CO_2_ incubator. NIH3T3 cells were seeded into a 96-well plate at a concentration of 1 ×10^4^ cells/well in 100 μL medium in triplicate for 24 h. Then, the medium was replaced by H_2_O_2_, Pd/Au NPs, Pd/Au NMs with different concentrations. 2 h later, 10 μL/well Cell Counting Kit-8 (CCK-8) was added after washing with PBS twice, allowing cells to continuously culture for 30min in the culture incubator. Absorbance at 450 nm was then measured in a BioTek SynergyHTX microplate reader. Live/dead staining has further assessed the biocompatibility of Pd/Au nanomotors by cell viability. The Jurkat T cells were seeded in a 96-well plate and incubated with nanomotors for 2 h. Subsequently, the treated cells were stained with Calcein-AM (green)/PI(red) for 30 min. The staining results of cells were observed under an inverted fluorescence microscope.

### Instruments

The morphology of the tested nanoparticles was captured with a transmission electron microscope (JEM-1400 Plus, JEOL) and scanning electron microscope (SEM) images were recorded on Field-Emission-SEM (Zeiss GeminiSEM500, Germany). Energy-dispersive X-ray spectroscopies (EDX) were obtained by elemental analysis with an EDX analyzer installed on a Field-Emission-SEM (Zeiss GeminiSEM500, Germany) at an accelerating voltage of 15kV. Nikon Ti2-A Inversion Fluorescence Microscope was utilized to track the motion of nanomotors, cell morphology, and fluorescence images. A Multi-Mode Reader (BioTek SynergyHTX, United States)was used to detect the absorbance of the CCK-8 assay.

## Results and Discussion

### Synthesis and Characterization of the Spiky Nanomotors

**FIGURE 2:**
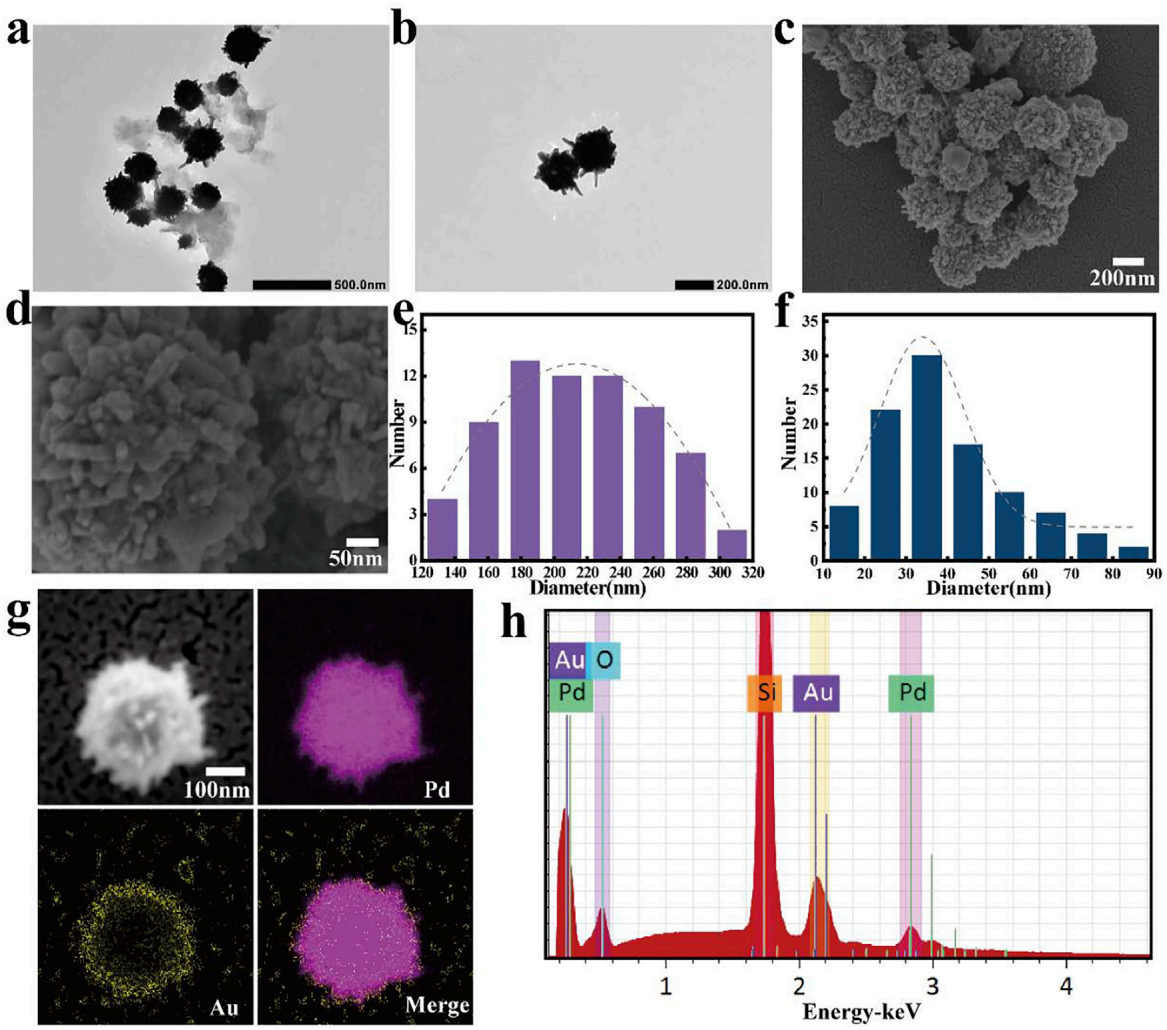
**(A,B)** TEM images of Pd nanospikes, scale bars were 500 and 200 nm, respectively; **(C,D)** SEM images of gold (Au)-coated Pd nanoparticles; scale bars were 200 and 50 nm, respectively; **(E)** Diameter distribution of Pd nanospheres; **(F)** Length distribution of the spikes dispersed on the surface of the nanospheres (The gray dash line is the corresponding Gaussian fitting curve); **(G)** Elemental mapping result of gold (Au)-coated Pd Janus nanomotor (scale bar, 100 nm) and **(H)** corresponding energy spectra (placed on a silicon substrate).

To synthesize Pd nanoparticles with dendritic structure, oleic acid (OA) and oleylamine (OAm) were used as surfactants/templates, and benzyl alcohol play as the solvent and reducing agent. First, OA and OAm were dissolved in benzyl alcohol to form spherical micelles having a dendritic peripheral structure. Then PdCl^4-^ ions were slowly reduced and deposited on the interior of the micelles and on the dendrites. Finally, the Pd nanoparticles with special morphology were obtained by removing the OA/OAm templates with ethanol washing. As depicted in Figures 2A,2B, TEM images, the as-prepared Pd nanoparticles have a 3D dendritic structure, which consists of spherical structures with diameters of about 215 ± 50 nm (Figure 2E) and dendrites with an average length of 40 ± 17 nm (Figure 2F) that are densely dispersed on the surface of the nanospheres.

For the formation of Pd/Au Janus structures, a layer of the spiky Pd nanoparticles was uniformly dispersed onto a glass slide, followed by an asymmetrical coating of the nanospheres by sputtering with a thin (5 nm) Au layer. The SEM images are shown in Figures 2C,D further confirms its unique structure. According to its corresponding energy-dispersive X-ray spectroscopy (EDX) (Figure 2H), demonstrating the presence of Pd and Au about 64%, 17%, respectively. In addition, Figure 2G is clearly shown that the Au element is distributed on surface of the Pd nanospheres. These results demonstrate the successful preparation of spiky-like nanomotors, indicating its geometrical asymmetry ensures an asymmetrical generation of forces.

### Motion Behavior of the Spiky Nanomotors

**FIGURE 3:**
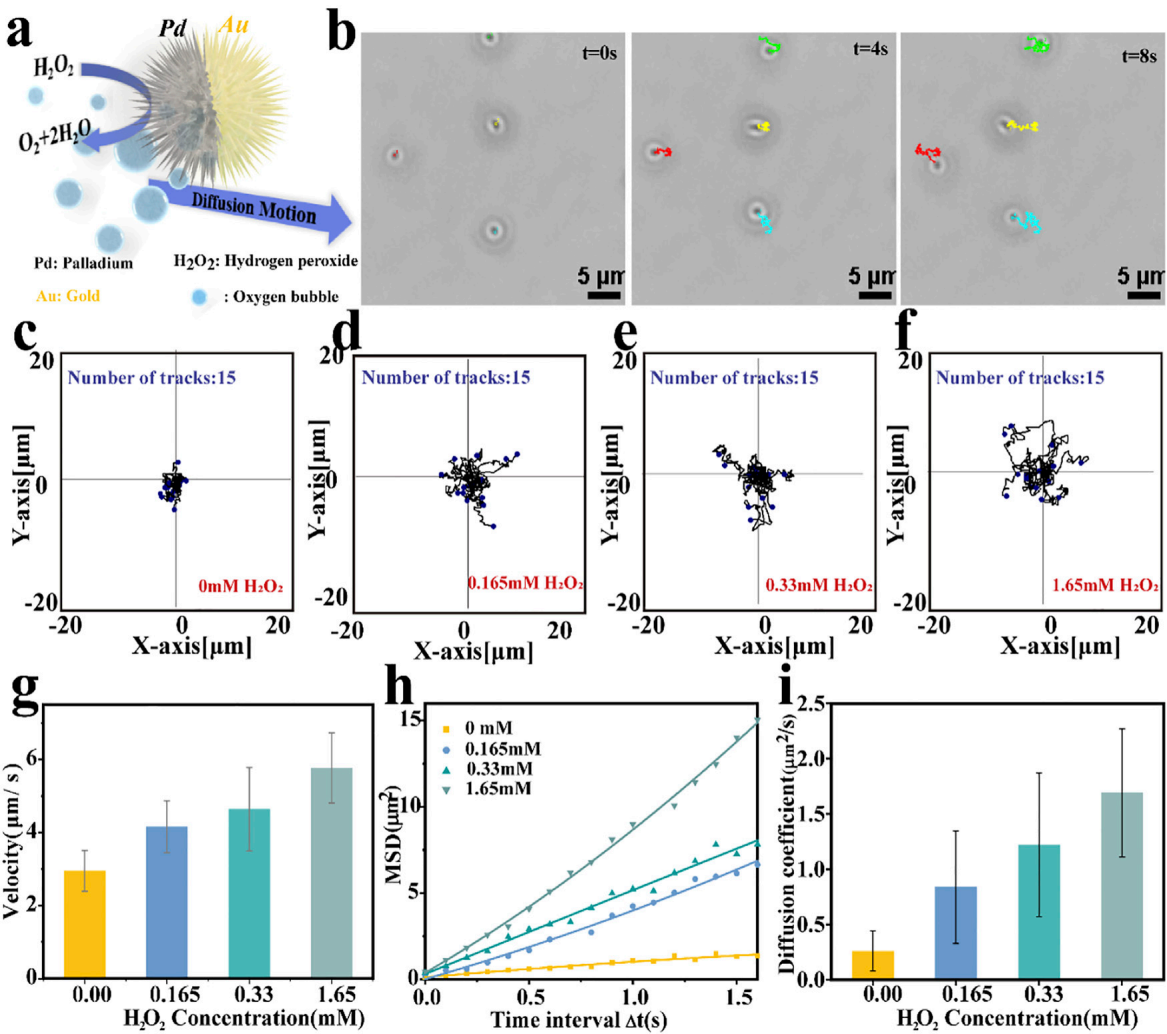
**(A)** Schematic diagram of the propulsion mechanism of spiky Pd/Au bimetallic nanomotors which depend on the decomposition of hydrogen peroxide. **(B)** Time-lapse images showing the movement of Pd/Au nanomotors in the presence of hydrogen peroxide fuel (0.165 mM H_2_O_2_) at 0,4,8 s, respectively; The scale bar was 5 μm. **(C–F)** Tracking paths (time interval = 100 ms, duration 10 s; 15 trajectories were analyzed) of Pd/Au nanomotor under different H_2_O_2_ concentrations (0.00 mM,0.165 mM,0.33 mM, 1.65 mM, respectively). **(G)** Average velocity of Pd/Au nanomotors at different fuel concentrations. **(H)** Mean square displacements (MSD)curve and **(I)** corresponding diffusion coefficients of Pd/Au nanomotors with different H_2_O_2_ concentrations.

After confirming the asymmetric structure of the nanomotor, its self-propelling ability was recorded by an inverted optical microscope and analyzed by the manual tracking plugin of ImageJ software according to previous reports. Owing to the Janus structure of Pd NPs coated with Au nano-sized thin, an effective propulsion driving force was generated through the asymmetric catalytic decomposition reaction of the hydrogen peroxide, as illustrated in Figure 3A. The optical tracking images are shown in Figure 3B (captured from Supplementary Video S2), illustrating the obvious autonomous propulsion of motors in an H_2_O_2_ solution. Varying the fuel concentrations, changing motion was observed, as the typical tracking trajectories displayed in Figures 3C–F (acquired from Supplementary Videos S1–S4). In the absence of fuel, the motion trajectory of the motor showed a typical random walk and localized within a small area behavior as a result of Brownian motion. As the fuel concentration increases, the catalytic nanomotors display relatively efficient propulsion. Obtained the coordinates of the moving trajectories and quantified the corresponding velocity of the motor by ImageJ on the basis of the optical tracking videos, results are depicted in Figure 3G. It is obvious that the velocity of the motor can be adjusted by the concentration of H_2_O_2_, the average velocity of the motor is 4.16 μm s^−1^ and 4.64 μm s^−1^at H_2_O_2_ concentrations of 0.165 and 0.33 mM, respectively. When the H_2_O_2_ concentration is up to1.65 mM, the nanomotors conducted self-driving motion with a speed of 5.77 μm s^−1^, which is 1.95 times higher than that of performing Brownian motion (2.95 μm s^−1^). Further, according to the X-Y coordinates of the trajectories, obtained from the optical videos using ImageJ software, the mean square displacements (MSD) of Pd/Au nanomotors along with different concentrations were investigate (You et al., 2019; Tang et al., 2020). As shown in Figure 2H, the plots of MSD versus time interval (t) reveal that the slope of MSD plots tends to increase with the concentration of the fuel. Subsequently, fitted the plots and estimated the effective diffusion coefficient (D_L_) according to formula 2. The corresponding results are shown in Figure 2I where the diffusion coefficient shows a significant increase from 0.26 ± 0.18 μm^2^ s^−1^ (without fuel) to 0.83 ± 0.51 μm^2^ s^−1^ (in the presence of 0.165 mM H_2_O_2_) and with a maximum enhancement of 5 times to 1.69 ± 0.58 μm^2^ s^−1^ (1.65 mM H_2_O_2_), exhibiting the fuel concentration dependence of enhanced diffusion motion. These results showed that the Pd/Au nanomotor system could achieve enhanced motion in the presence of hydrogen peroxide even under a relatively low concentration, providing the possibility for subsequent application in the bioenvironment.

### T Cell Activation Ability of the Spiky Nanomotors

**FIGURE 4:**
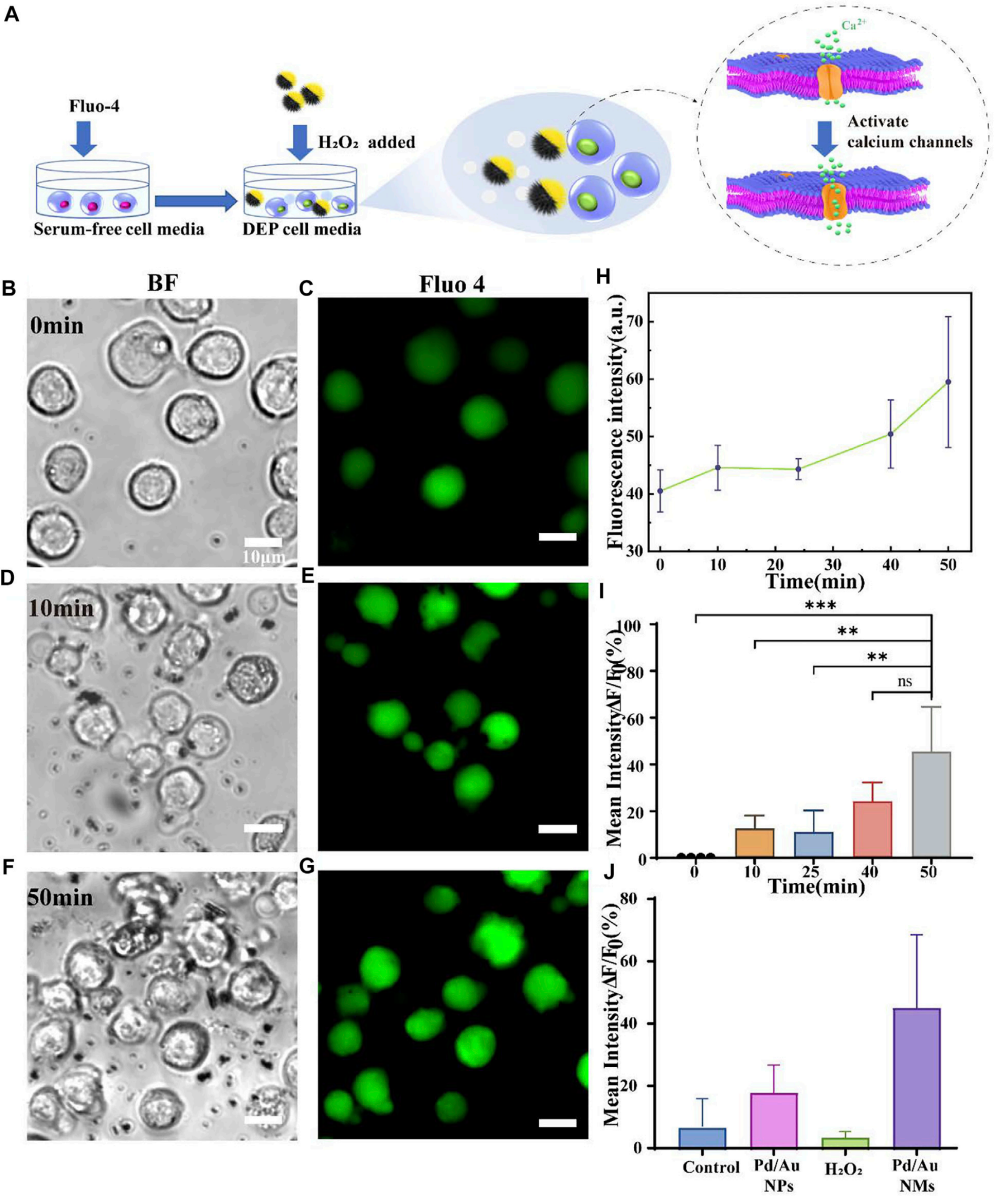
**(A)** Schematic illustration of the spiky Janus nanomotors to activate T cells *via* the calcium-permeable ion channel. **(B,D,F)** Bright-field and **(C,E,G)** corresponding fluorescent images showing the interaction between T cells and nanomotors at *t* = 0,10, and 50 min, respectively. Scale bars = 10 μm; **(H)** Plotted the fluorescence intensity against time is to show the calcium response of Jurkat T cells when incubated with Pd/Au nanomotors (*n* = 4); **(I)** the corresponding increment of calcium fluorescence intensity over time (relative to the initial time, *t* = 0 min). The asterisk (*) denotes that the statistical significance between groups (**p < 0.01, ***p < 0.001); **(J)** Comparison of the increase in fluorescence intensity among different groups within the same time interval (50 min): the resting Jurkat T cells, cells with Pd/Au nanomotors (Pd/Au NMs, 0.165 mM H_2_O_2_), Pd/Au nanoparticles (Pd/Au NPs) and hydrogen peroxide (0.165 mM H_2_O_2_).

To address whether the moving Pd/Au nanomotors could optimize the activation of the immune cells *via* mechanotransduction, we monitored the performance to activate Jurkat T cells, which express high levels of endogenous mechanical sensitive ion channel (Liu and Ganguly, 2019). The Jurkat cell line has been used to model and characterize signaling events in T cell activation (TCA), a critical process in an effective adaptive immune response (Abraham and Weiss, 2004). To prove that spiky nanomotors can achieve high-efficiency movement in biologically relevant media, the self-driving ability of the nanomotors has been observed in the dielectrophoresis (DEP)cell culture medium (Supplementary Video S5). Note that the DEP medium is a non-ionic iso-osmotic solution with a small dielectric shielding effect, so the effect of the background solution on the motor movement efficiency can be eliminated to a greater extent (Marino et al., 2015). Considering high concentration of hydrogen peroxide is cytotoxic, so we chose a relatively low concentration of 0.165 mM that does not harm the cells and also has a relatively high speed of motion as the driving fuel (Supplementary Figure S8) (Kuang et al., 2011; Yu et al., 2021). As shown in Supplementary Figure S1 (Supplementary Material), in the presence of 0.165 mM hydrogen peroxide, the nanomotor can approach the cells or move continuously in the culture medium. Then, to quantify T cell activation, we measured the concentration fluctuation of intracellular Ca^2+^ ions over the duration of the operation of the nanomotors. It has been verified that professional mechanical sensitive ion channels (especially Piezo 1 ion channels) are calcium ion-permeable and the activation of such channels is accompanied by an influx of Ca^2+^ into the cell cytosol from the extracellular environment (Pottosin et al., 2015; Ranade et al., 2015; Liu et al., 2018; Atcha et al., 2021). Also, a rapid increase of calcium ion concentration in the cytoplasm is a common mark of T cell activation, this suggested that mechanical-mediated T cell activation could be reflected *via* the dynamic change of calcium flux (Dirar et al., 2020). In our experiments, the influx of Ca^2+^ was measured by a calcium-sensitive dye, Fluo-4, which was loaded into the cell cytosol. The fluorescence emission of Fluo-4 increases proportionally to intracellular [Ca^2+^], so it provides a direct and quantitative readout of the T cell activation (Figure 4A). Considering the oscillation and flow of the entire solution system caused by the movement of the autocatalytic motor, the state of the whole cell was observed. The images recorded in Figures 4B–G illustrate the effect of the moving nanospikes on the overall cells (*t* = 0 min, *t* = 50 min) and then Image J analysis was used to obtain the fluctuation results of calcium ion concentration. It displays that the mean values of the Ca^2+^ fluorescence intensity gradually increased, and reaches the maximum value at *t* = 50 min (Figure 4H). Compare their fluorescence intensity at *t* = 50 min, the intensity of the former is 1.45 ± 0.19 times that of the resting T cells (*t* = 0 min) (Figure 4I). In the same time interval, resting T cells only containing Fluo-4 exhibited a 7.12 ± 7.13% increase in fluorescence (Supplementary Figure S3). To exclude the influence of chemical fuel hydrogen peroxide on the state of cells, calcium ions were tested under the same conditions, as depicted in Figure 4J, and an evaluation of the corresponding results implies that the interference of low concentration hydrogen peroxide solution (0.165 mM) can be ignored (an increase of 4.08 ± 1.34%). Moreover, to explore the performance of the unique self-driving character of the nanomotor on the activation efficiency, we incubated the spiky-like nanoparticles with Jurkat T cells without adding hydrogen peroxide as fuel and examined calcium fluorescent intensity after the same time interval (Figure 4J). Compared with movable nanomotors, static nanoparticles influence T cell activation with an amplitude (1.18 ± 0.07 fold) smaller than the dynamic state (1.45 ± 0.19 fold). In addition to the effective self-driving force of the dynamic motor, the dynamic behavior of the nanomotor in the cell microenvironment induces fluid flows, generating distinct flow fields surrounding the cell is also an important physical factor, according to reports in the literature (Syeda et al., 2015; Liu et al., 2018). Working synergistically with the inherent nature of the motor to enhance the forces exerted on the cell during activation. Sustained acts for a period of time, mechanosensitive ion channels on T cells percept these physical stimuli and mediate an intracellular calcium response, which contributes to the activation of Jurkat T cells in the overall environment.

### Evaluate the Mechanical Force Generated by a Single Nanomotor

The self-propulsion of the nanomotors endows itself as an effective force. To calculate how large the force is, we based on a theory for self-propelled particles, involving the long-time translational diffusion coefficient reported by ten Hagen et al. (2011) (ten Hagen et al., 2015). Such aspherical particles with two rotational degrees of freedom, the absolute value of the effective driving force can be evaluated by the following formula 3:

$$F=\frac{3k_{B}T}{2r}\sqrt{2\left(\frac{D_{L}}{D_{\tau }}-1\right)}$$

where k_B_ is the Boltzmann constant, T the absolute temperature, r the radius of the nanoparticles, and D_L_, D_τ_ is the long-time and translational diffusion coefficient, respectively.

In our cases, the active motion of the self-propelled Janus nanomotors was investigated by mean square displacement (MSD) analysis (Figure 2I). In the absence of fuel, Pd/Au nanomotors perform Brownian motion, the value of D_L_ is equal to the translational diffusion coefficient Dτ (Campbell and Ebbens, 2013; Ma X. et al., 2015; You et al., 2019). As illustrated in Figure 2I, the value of Dτ was about 0.26 ± 0.18 μm^2^ s^−1^, at room temperature of 25°C. At 0.165 mM H_2_O_2_, the diffusion coefficient is increased by 2.13 times compared to that without fuel. Taken together, we can estimate the effective driving force generated by a single nanomotor with the decomposition of 0.165 mM hydrogen peroxide is about 120.94 ± 79.6 fN, which is approximate to that calculated by DLS results (Supplementary Figure S6).

In addition, the dynamic behavior of multiple nanomotors in the cellular microenvironment causes rapid local fluid convection, generating pressure and shear stresses around the cell, thereby synergizing the forces of multiple nanomotors and enhancing the expression of mechanical signals to a certain extent (Takatori et al., 2014). Mechanosensitive ion channels, particularly the Piezo1 ion channel used as a physical stimulus sensor, have recently been shown to be abundantly expressed on T cells (Liu and Ganguly, 2019). The mechanical force or shear stress produced by the advancing nanospike is captured by such biosensors and benefits ion influx or efflux, which is a prerequisite for subsequent T cell activation (Syeda et al., 2015; Liu et al., 2018).

## Conclusion

In summary, novel bionic spiky Pd Janus nanomotors were successfully fabricated. Driven by the catalytic reaction of hydrogen peroxide at low concentrations, we further estimated the corresponding driving force and explored their activation effect on Jurkat T cells *in vitro*. Further, the fluid convection caused by the locomotion property of nanomotors in the biological microenvironment generated local pressure and shear stress around the cells cooperating with the force and inherent characteristics of the nanomotors to enhance the mechanical signal. The nanomotor system represents a platform to achieve local regulation of T cells through physical signals, which is different from T cell activation in cytokines ways, risking the excessive activation of T cells and immunological storm. Additionally, benefiting from the special dendritic structure on the motor surface enhances the adhesion ability and also promotes the mechanical stress of the motor on the cell membrane. An intracellular calcium response mediated by the mechanosensitive ion channels optimizes the activation of T cells through physical effects. Activation of T cells is a critical step in immunotherapy, the current research not only provides a new way for the application of the special properties of motors in cancer immunotherapy but also further clarifies the significance of mechanical force signaling in T cell activation.

## Data Availability

The original contributions presented in the study are included in the article/Supplementary Material, further inquiries can be directed to the corresponding author.
