# Supplementary material for: Mechanically Optimize T Cells Activation by Spiky Nanomotors
Source: Front Bioeng Biotechnol. 2022 Feb 22;10:844091. doi: 10.3389/fbioe.2022.844091 (PMC8902353; doi:10.3389/fbioe.2022.844091)
Supplement: Supplementary file 6 [file DataSheet1.docx]

Supplementary Material

# Supplementary Figures


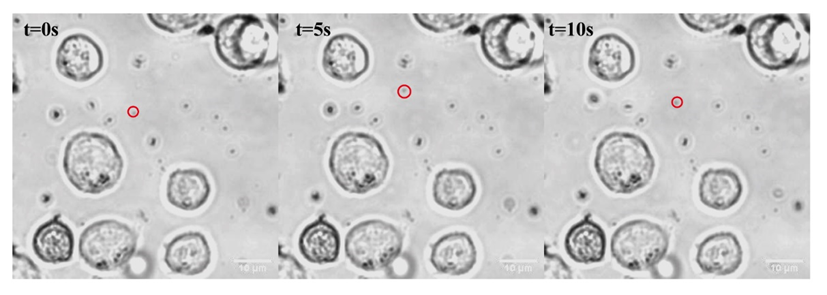


**Supplementary Figure 1.** Time-lapse images of Pd/Au nanomotors moving in a cell media with 0.165mM H_2_O_2_ added (captured from Supporting video5).

**
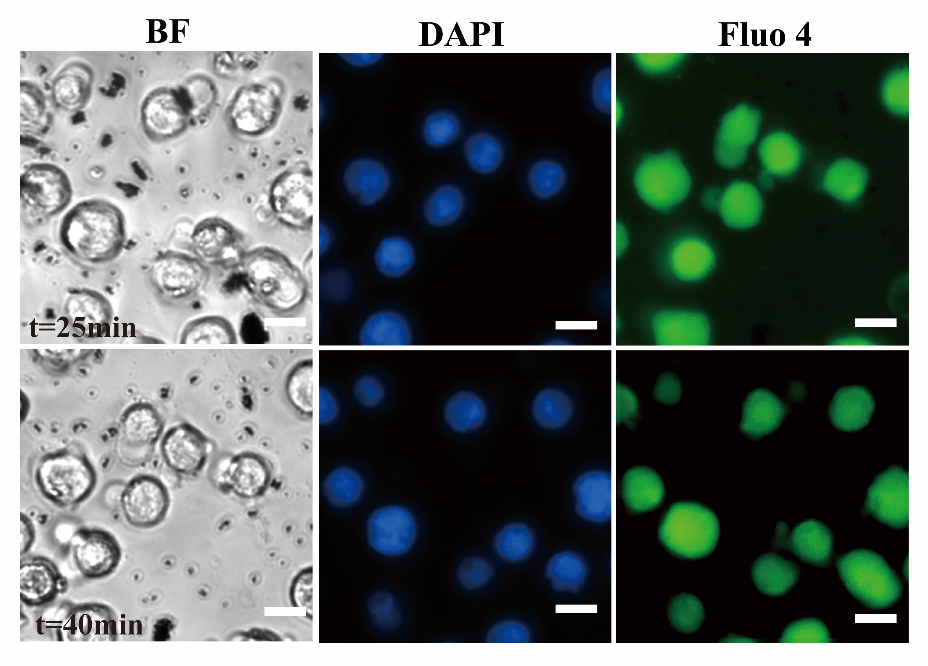
**

**Supplementary Figure 2.** Bright-field and corresponding fluorescent images of Jurkat T cells stimulated by Pd/Au nanomotors with fuel added (0.165 mM H_2_O_2_) at t = 25 min, and t = 40min, respectively. Scale bars=10 µm.


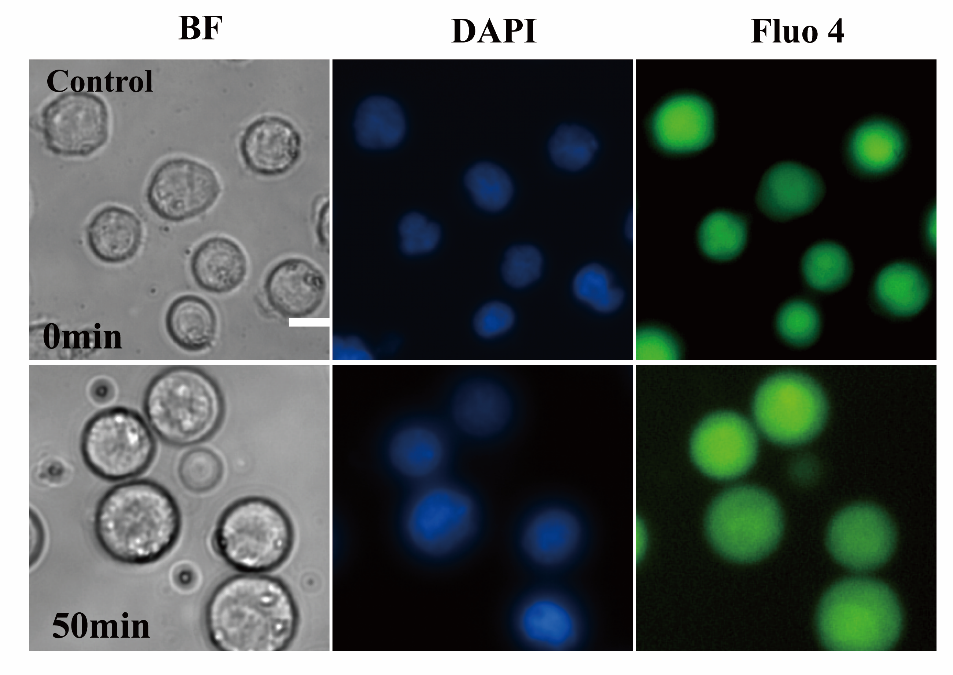


**Supplementary Figure 3.** Bright-field and fluorescence images of Jurkat T cells of blank controls lasting the same time interval (t=50min).


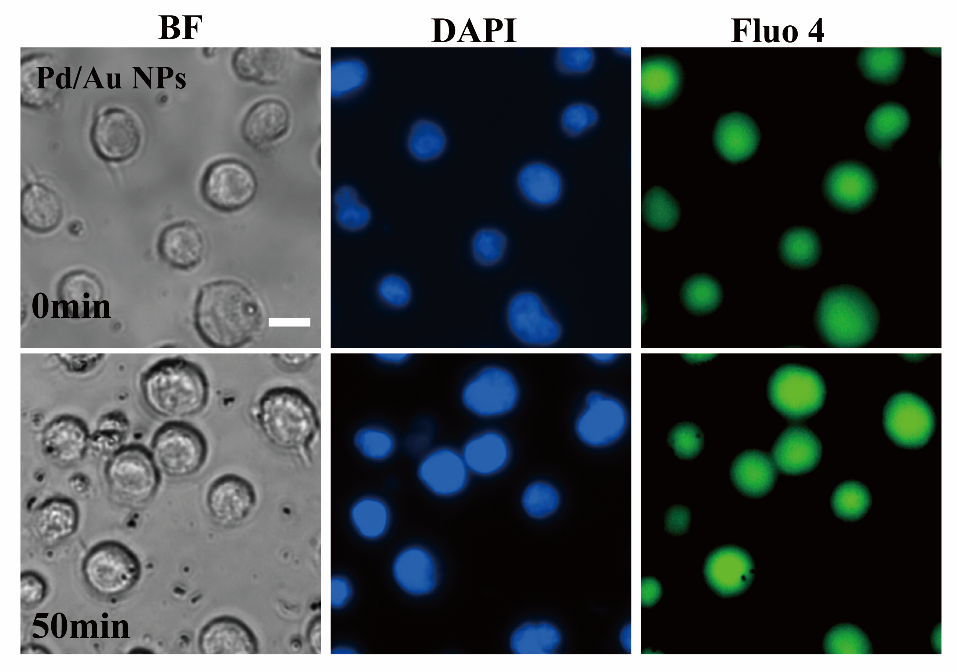


**Supplementary Figure 4.** Bright-field and fluorescence images of Jurkat T cells incubated with Pd/Au nanoparticles(Pd/Au NPs) for the same time interval (t=50min).


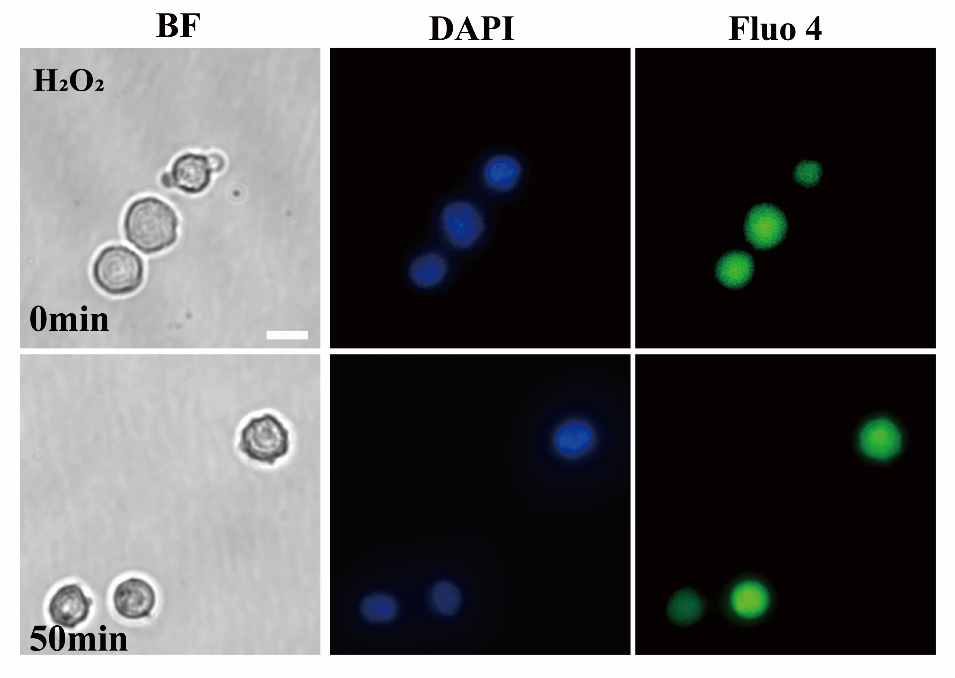


**Supplementary Figure 5.** Bright-field and fluorescence images of Jurkat T cells incubated with the driving fuel (0.165mM hydrogen peroxide) for the same time interval (t=50min).

**Supplementary Figure 6.** Size distribution of Pd/Au nanospikes measured by dynamic light scatter(DLS). Number mean=226.7±30 nm, PDI=0.217, diffusion coefficient (Dτ)=0.37±0.08 µm^2^s^-1^.

**Supplementary Figure 7.** The effective driving force was calculated by analyzing the MSD (blue) and by DLS (gray) of Pd/Au nanomotors with different H_2_O_2_ concentrations.





**Supplementary Figure 8.** Cell Viability of NIH 3T3 cells incubated with different concentrations of hydrogen peroxide for 2 h.





**Supplementary Figure 9.** Cell Viability of NIH 3T3 cells incubated with different concentrations of Pd/Au nanoparticles (Pd/Au NPs) for 2 h.





**Supplementary Figure 10.** Cell Viability of NIH 3T3 cells incubated with Pd/Au nanomotors(in the presence of 0.165mM hydrogen peroxide) for 2h,4h,8h.


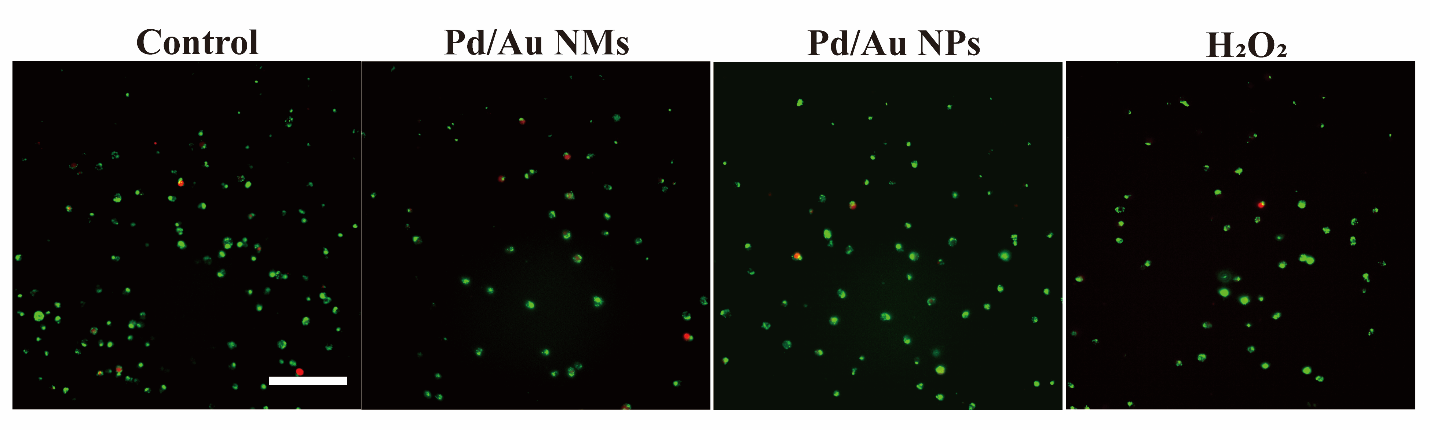


**Supplementary Figure 11.** Live/dead staining of Jurkat T cells incubated with different groups for 2 hours. Live cells are stained with AM (green), and dead cells are stained with PI (red). Scale bar, 100 μm.

# Supplementary Videos

Video S1. Brownian motion of Pd/Au nanomotors

Video S2. Movement of Pd/Au nanomotors in 0.165mM hydrogen peroxide solution

Video S3. Movement of Pd/Au nanomotors in 0.33mM hydrogen peroxide solution

Video S4. Movement of Pd/Au nanomotors in 1.65mM hydrogen peroxide solution

Video S5. Movement of Pd/Au nanomotors (with 0.165mM hydrogen peroxide added) in cell culture media

**
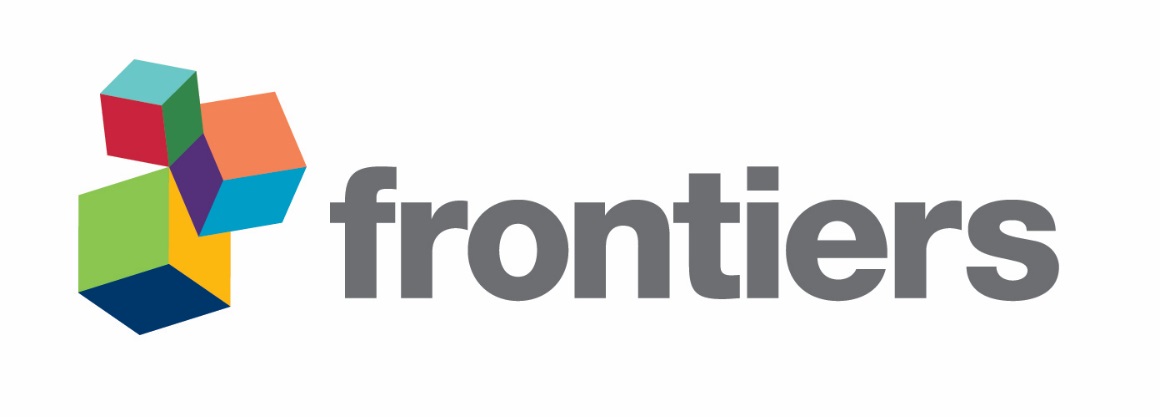
**
